# The Emergence of AI-Based Wearable Sensors for Digital Health Technology: A Review

**DOI:** 10.3390/s23239498

**Published:** 2023-11-29

**Authors:** Shaghayegh Shajari, Kirankumar Kuruvinashetti, Amin Komeili, Uttandaraman Sundararaj

**Affiliations:** 1 Center for Applied Polymers and Nanotechnology (CAPNA), Department of Chemical and Petroleum Engineering, University of Calgary, Calgary, AB T2N1 N4, Canada; shaghayegh.shajari@northwestern.edu; 2 Center for Bio-Integrated Electronics (CBIE), Querrey Simpson Institute for Bioelectronics (QSIB), Northwestern University, Evanston, IL 60208, USA; 3 Intelligent Human and Animal Assistive Devices, Department of Biomedical Engineering, University of Calgary, Calgary, AB T2N 1N4, Canada; kirankumar.kuruvinas@ucalgary.ca (K.K.); amin.komeili@ucalgary.ca (A.K.); 4 Department of Mechanical and Manufacturing Engineering, University of Calgary, Calgary, AB T2N 1N4, Canada

**Keywords:** wearable sensors, physical sensors, chemical sensors, biosensors, personalized health monitoring, disease diagnosis and monitoring, intelligent sensing, artificial intelligence (AI), machine learning (ML), deep learning (DL)

## Abstract

Disease diagnosis and monitoring using conventional healthcare services is typically expensive and has limited accuracy. Wearable health technology based on flexible electronics has gained tremendous attention in recent years for monitoring patient health owing to attractive features, such as lower medical costs, quick access to patient health data, ability to operate and transmit data in harsh environments, storage at room temperature, non-invasive implementation, mass scaling, etc. This technology provides an opportunity for disease pre-diagnosis and immediate therapy. Wearable sensors have opened a new area of personalized health monitoring by accurately measuring physical states and biochemical signals. Despite the progress to date in the development of wearable sensors, there are still several limitations in the accuracy of the data collected, precise disease diagnosis, and early treatment. This necessitates advances in applied materials and structures and using artificial intelligence (AI)-enabled wearable sensors to extract target signals for accurate clinical decision-making and efficient medical care. In this paper, we review two significant aspects of smart wearable sensors. First, we offer an overview of the most recent progress in improving wearable sensor performance for physical, chemical, and biosensors, focusing on materials, structural configurations, and transduction mechanisms. Next, we review the use of AI technology in combination with wearable technology for big data processing, self-learning, power-efficiency, real-time data acquisition and processing, and personalized health for an intelligent sensing platform. Finally, we present the challenges and future opportunities associated with smart wearable sensors.

## 1. Introduction

The healthcare industry faces a significant shift towards digital health technology, with a growing demand for real-time and continuous health monitoring and disease diagnostics [1,2,3]. The rising prevalence of chronic diseases, such as diabetes, heart disease, and cancer, coupled with an aging population, has increased the need for remote and continuous health monitoring [4,5,6,7]. This has led to the emergence of artificial intelligence (AI)-based wearable sensors that can collect, analyze, and transmit real-time health data to healthcare providers so that they can make efficient decisions based on patient data. Therefore, wearable sensors have become increasingly popular due to their ability to provide a non-invasive and convenient means of monitoring patient health. These wearable sensors can track various health parameters, such as heart rate, blood pressure, oxygen saturation, skin temperature, physical activity levels, sleep patterns, and biochemical markers, such as glucose, cortisol, lactates, electrolytes, and pH and environmental parameters [1,8,9,10]. Wearable health technology includes first-generation wearable technologies, such as fitness trackers, smartwatches, and current wearable sensors, and is a powerful tool in addressing healthcare challenges [2].

The data collected by wearable sensors can be analyzed using machine learning (ML) and AI algorithms to provide insights into an individual’s health status, enabling early detection of health issues and the provision of personalized healthcare [6,11]. One of the most significant advantages of AI-based wearable health technology is to promote preventive healthcare. This enables individuals and healthcare providers to proactively address symptomatic conditions before they become more severe [12,13,14,15]. Wearable devices can also encourage healthy behavior by providing incentives, reminders, and feedback to individuals, such as staying active, hydrating, eating healthily, and maintaining a healthy lifestyle by measuring hydration biomarkers and nutrients. In addition, they can significantly reduce healthcare costs by reducing the number of hospitalizations and emergency room visits, which can be expensive for both individuals and the healthcare system. Wearable technology enables remote monitoring of patients and personalized care. Further recommendations will be available to the patients through AI algorithms on an individual’s diet and exercise habits. These tailored suggestions aim to improve overall health and reduce the risk of chronic diseases. [4,14,16].

Despite the potential benefits of wearable health technology, there are several challenges, such as the accuracy and reliability of the data collected by these devices. As the biosensors are still in the development stage in monitoring biochemical signals, the technology is yet to sense biological biomarkers continuously, and they need to achieve higher sensitivity, selectivity, and accuracy towards specific biomolecule sensing [14,17]. There is still a risk of inaccurate readings, which can lead to false alarms or missed opportunities for early detection of health issues. Therefore, it is necessary to validate the technology with gold standard methods, such as blood chemistry and other relevant standard techniques. Another challenge is that of privacy and security concerns [18]. When using wearable technology to collect and transmit sensitive health data, there is a risk of data breaches and unauthorized access to personal information. Healthcare providers and device manufacturers must ensure appropriate and extra care [4].

Integration of AI is revolutionizing the accuracy and efficiency of wearable sensors by identifying and correcting errors in the collected data. For instance, inaccuracies in heart rate data can be rectified, ensuring the reliability and precision of the devices [16,19]. AI algorithms can be used to analyze the massive amount of data collected by wearable devices, enabling healthcare providers to identify patterns, predict health outcomes, and make knowledgeable decisions about patient care. For instance, by examining an individual’s activity level, sleep patterns, and heart rate, AI algorithms can forecast the likelihood of heart attacks or strokes, empowering healthcare providers to take proactive measures [8,20].

Moreover, when it comes to multimodal sensing and cross-sensitivity issues where the measurement of one signal is influenced by the presence of other signals, AI pattern recognition models can be employed. These models are trained to identify specific patterns associated with each signal, enabling the isolation of individual signal contributions even in the presence of cross-sensitivity. For instance, an ML-based multimodal electrochemical analytical device, utilizing eMoSx-Laser Induced Graphene, was used for the multiplexed detection of tyrosine and uric acid in sweat and saliva [21]. The electrodeposition of MoSx resulted in an increased electrochemically active surface area and heterogeneous electron transfer rate constant. Features extracted from the electrochemical data were utilized to train ML models for predicting analyte concentrations in both single spiked and mixed samples. In addition, advanced signal processing techniques, coupled with AI models, can be applied to separate and extract relevant information from mixed signals [22]. Additionally, AI filtering techniques can address the cross-sensitivity of multiple biomarker sensing, contributing to more accurate and reliable measurements in healthcare applications [23]. Moreover, it was shown that a deep neural network (DNN) for the multiplex detection of single particles and molecular biomarkers can be employed [24]. The AI model could integrate rapid wavelet particle detection with short-time Fourier Transform analysis, followed by DNN identification on a specialized AI device. This approach was validated through the multi-spot optical excitation of Klebsiella Pneumoniae bacterial nucleic acids flowing through an optofluidic waveguide chip, generating fluorescence signals with varying amplitude, duration, and quality.

Another critical challenge in wearable sensor networks is managing their energy consumption, particularly when using wireless sensor networks (WSN), due to the often non-replaceable or rechargeable nature of sensor batteries. Efficient energy usage is deemed imperative, necessitating a thoughtful and optimal design of the WSN and sensor routing [25]. In particular, when using multiple sensors, traffic uncertainty poses a challenge by the variability in data generation rates from the body sensors [25]. In this context, a wireless body area sensor network (WBSN) is designed based on one or more sinks to enable biosensors to first transmit the data to these sinks for storage, processing, and transmission to another network [25]. Another technique is using a repulsion-based improved grey wolf optimizer for the sensor nodes localization that outperforms the traditional Global Positioning System [26]. Alternatively, AI plays a pivotal and significant role in optimizing the design and management of WBSNs in the healthcare sector [27]. AI and ML-based time series analysis can perform comprehensive, and time-synchronized data collection from the body sensor network framework and integrate real-time location systems [27]. ML techniques, such as signal processing algorithms, facilitate accurate interpretation of physiological data collected by body sensors, while energy-efficient routing algorithms leverage reinforcement learning to adaptively optimize network pathways based on real-time energy constraints [28]. Predictive maintenance models could utilize AI to anticipate sensor failures, and dynamic resource allocation algorithms adjust bandwidth and power allocations dynamically [27].

In summary, this review paper explores the emergence of AI-based wearable sensors for digital health technology. We discuss the types of wearable sensors, including physical, physiological, and chemical sensors, their sensing materials, structures, and mechanisms, and their applications in human health monitoring. In addition, the challenges and opportunities associated with the use of wearable sensors in healthcare and the potential implications for the future of healthcare with the advancement of AI are also covered. Overall, this review paper aims to provide a comprehensive overview of the current state of AI-based wearable sensors in healthcare and their potential impact on the healthcare industry.

## 2. Overview of Wearable Health Technology

### 2.1. Types of Wearable Health Sensors

**Figure 1:**
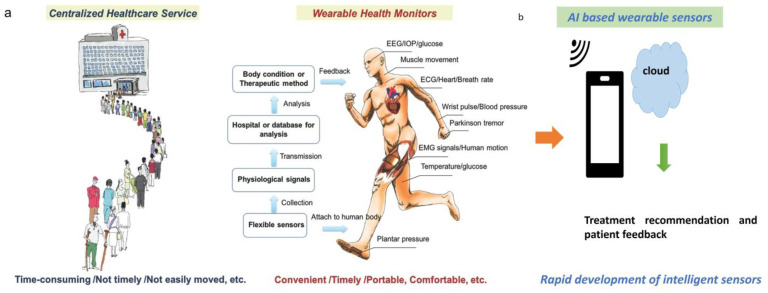
(**a**) Wearable health monitoring versus centralized healthcare services as a convenient method in remote and real-time personalized healthcare systems. Reproduced with permission from [34]. (**b**) Artificial intelligence (AI) tools and personalized health monitoring toward treatments and disease diagnosis.

Wearable sensors for human health monitoring can be classified into physical sensors for measuring physical and physiological signals, chemical sensors, and biosensors for measuring chemical signals [13]. Wearable sensors are helping clinical and healthcare services with the possible shift from centralized clinical diagnostics to more individualized and personalized home care [29,30] (Figure 1a). With the help of wireless systems and AI, these wearable smart sensors can be personalized and be accessible to patients anywhere and anytime [31,32] (Figure 1b). They include a variety of wearable sensors that function in physical health conditions, such as real-time human motion activities, and physiological conditions (e.g., heart rate, blood pressure, and blood flow, body temperature, and hydration) [33], electrophysiological monitoring (e.g., ear, brain and muscle tissue activities) [34,35], and biosensing (e.g., glucose and Parkinson tremor) [36,37].

#### 2.1.1. Physical and Physiological Sensors

Physical sensors are electromechanical sensors that detect small-scale pressures and motions, such as subtle touch and heart pulse, and large-scale movements, such as human joint motions [2]. Therefore, they can be classified as pressure and strain sensors. Strain sensors convert mechanical deformations to electrical signals by measuring capacitance or resistance change [38]. High flexibility and stretchability are the essential features for the next generation of strain sensors. Recent progress in flexible devices focusing on electronic skin (E-skin) applications shows a requirement for stretchable and sensitive strain sensors that can tolerate over 30% strain [39]. Ultra-sensitive, flexible, and stretchable pressure sensors are also considered ideal for wearable health monitoring applications, making them essential components in e-skins.

Polymer nanocomposites have gained tremendous attention in recent stretchable smart electronics [40,41,42]. They are composed of conductive nanomaterials and an elastomeric matrix. An elastomer is one class of polymer that exhibits high elastic deformations of up to 1000%, and upon, or even beyond, unloading the strain/load, they can return to their original position [43]. Different type of polymer includes silicon elastomers (e.g., polydimethylsiloxane (PDMS) [44] and Ecoflex [45]), rubbers (e.g., natural rubber [46]), thermoplastic elastomers [47] and block copolymers (e.g., poly-(styrene-block-butadiene-block-styrene) [48]), fluoropolymers (e.g., polyvinylidene fluoride (PVDF) [49] and ethylene tetrafluoroethylene [50]), and fluoro-elastomers (e.g., FKM [51]), which have been used for strain sensing approaches. The conductive nanomaterials are classified as carbon- based nanofillers, such as carbon blacks (CBs) [52], carbon nanotubes (CNTs) [51] and graphene [53], and metallic-based nanofillers, such as silver nanowires (AgNWs) [54], copper nanowires [55], silver nanoparticles (AgNPs) [56], and gold nanoparticles (AuNPs) [57].

**Figure 2:**
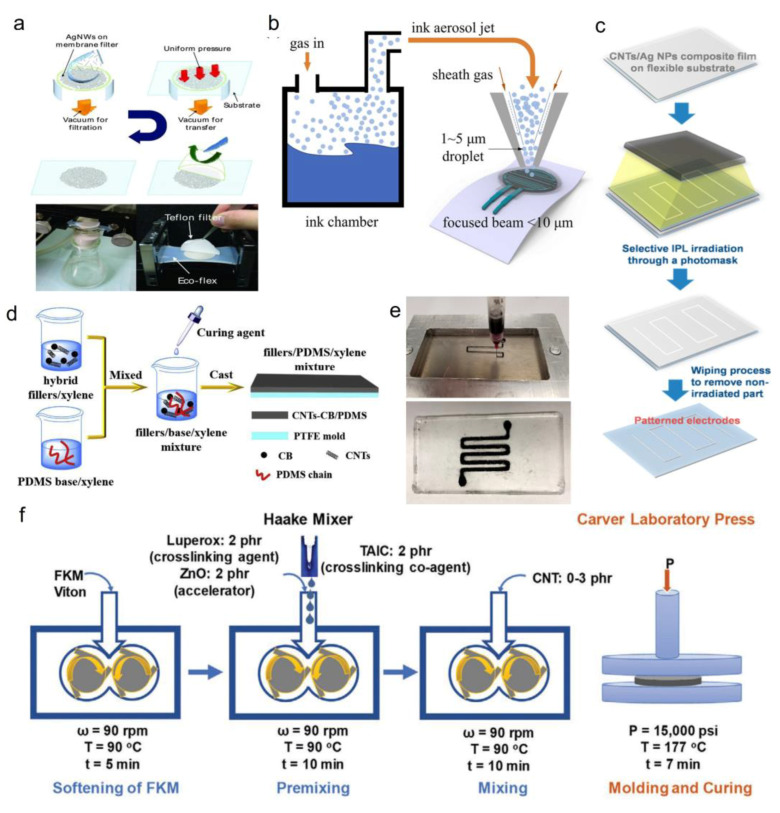
Different fabrication methods for flexible conductors: (**a**) Filtration method; transferring AgNW thin films from membrane filter on a pre-strained Ecoflex substrate. Reproduced with permission from [89]. (**b**) Printing method: jet printing of nanomaterial ink including silver nanoparticles and CNTs on the top of flexible substrate. Reproduced with permission from [90]. (**c**) Micropatterning technique: selective patterning on a flexible substrate with CNTs/AgNPs composite mixture using intense pulsed light (IPL) irradiation and a photo mask. Reproduced with permission from [70]. (**d**) Solution blending: solution mixing of hybrid nanofiller–polymer nanocomposites. Reproduced with permission from [61]. (**e**) Printing method: 3D printing of uncured polymer and CNT solution inside a flexible PDMS. Reproduced with permission from [67]. (**f**) Melt-mixing method: Synthesis of CNT/FKM nanocomposites. Reproduced with permission from [73].

There are several processing methods for stretchable conductors, which can be classified into solution blending, melt mixing, and in situ polymerization [43,58]. Solution blending methods for fabrication of stretchable sensors and flexible conductors include solution casting [59,60], solution molding [51,61,62], spray and spin coatings [44,63,64], wet spinning [65,66], ink-jet and 3D printing [67,68], micropatterning [69,70], and filtration methods [69,71]. Various melt mixing techniques include extrusion [72], internal mixing [73,74,75], and two-roll mill mixing [46]. Figure 2 demonstrates different solution processing and melt mixing methods for fabricating various flexible conductors.

##### Strain/Pressure Sensing Mechanisms

Strain and pressure sensors have been widely used in instrumenting stand-alone testing devices to study the mechanical response of musculoskeletal systems at multi scales [76], as well as wearable devices, including orthoses [77], shoes [78], and flexible electronics [42]. The strain/pressure sensing mechanisms are piezoresistive [73,79], piezo capacitive [80,81], piezoelectric [82,83], triboelectric [84,85], and fiber Bragg grating sensors [86,87]. These mechanisms derive from alternations in the geometry’s changes, micro-/macro-structural changes, and intrinsic material properties. Strain sensing mechanisms in conventional strain sensors based on metals relied on geometrical changes. In semiconductors, such as silicon and germanium, the sensing mechanism is related to electron and hole transport in crystalline structures under strain and temperature effects [88].

**Figure 3:**
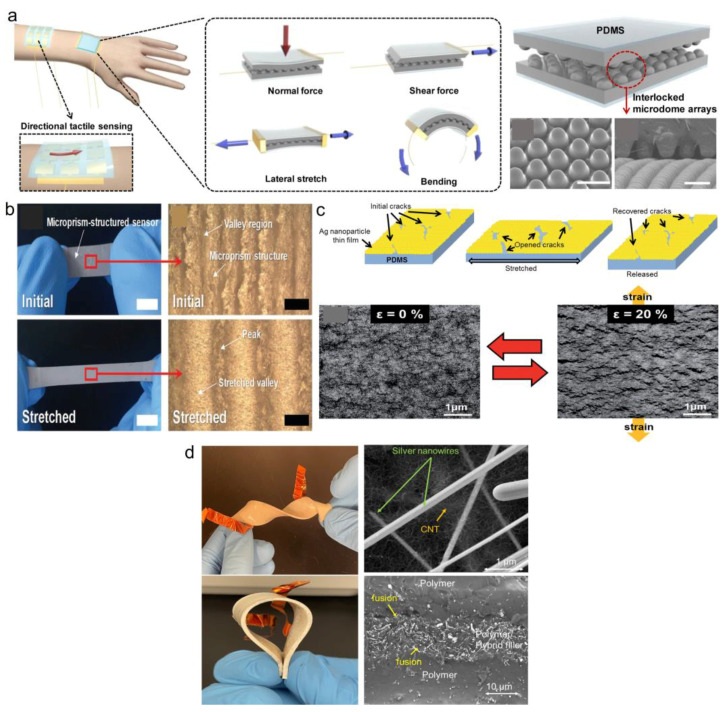
Disconnection mechanism in strain sensors with (**a**) interlocking structure in an electronic skin based on carbon nanotube-poly (dimethyl-siloxane) (CNT-PDMS) composite films with interlocked micro-dome arrays for a stress-direction detection and differentiation of various mechanical stimuli—reproduced with permission from [96]; (**b**) microprism structure: the valley and peak structures in a microstructured AgNW/PDMS composite film based stretchable strain sensor in the initial and stretched states—reproduced with permission from [98]; (**c**) crack propagation structure: formation of micro-cracks on Ag NP on PDMS substrate during annealing process and an elongation/relaxation cycle at 0% and 20% strain, which shows the number of micro-cracks opened at 20%, as compared to the relaxation status of the sensor—reproduced with permission from [103]; (**d**) overlapped filler networks of AgNWs and CNTs in a hybrid shell structure on flat flexible—reproduced with permission from [54].

The piezoresistive effect relates to the resistance change in response to physical deformation. Three main factors contribute to the resistance change in polymer composites with fillers: (a) loss of contact between neighboring fillers leading to disconnection mechanism, (b) alternations in tunneling distance between adjacent fillers relative to the tunneling mechanism, and (c) deformations of fillers, altering their intrinsic piezoresistive properties [91,92]. The disconnection and tunneling mechanisms are the dominant mechanisms [93,94], due to poor stress transfer from the polymer matrix to the fillers [95]. The disconnection mechanism can occur in different structures: (a) interlocking structures [96,97], in which any loss of contact between interlocking conductors changes the resistance dramatically (Figure 3a), (b) microprism structure consisting of peaks and valleys [98] or pre-strained substrate and by sliding the nanowire network upon stretching (Figure 3b), (c) crack propagation in filler network [99,100], which several cracks and disconnections initiate in the rigid filler network and which propagates upon stretching, but can be recovered by reconnecting the cracks after unloading (Figure 3c), (d) tunneling mechanism through overlapped filler networks, such as overlapped AgNWs and CNTs on flat flexible [101,102] (Figure 3d).

The piezo-capacitive effect under stretching or pressure is due to dimensional changes in the dielectric and substrate layer between two electrodes in the area and changes in thickness [80]. Piezoelectricity is an intrinsic property of materials, such as inorganic piezoelectric materials, including lead zirconate titanate, zinc oxide, barium titanate, aluminum nitride, lithium niobite, and quartz, and organic ones, such as PVDF, poly(L-lactic acid), and poly(D-lactic acid). However, the piezoelectric output of organic materials is much less than that of its inorganic counterparts. By applying strain or compression, a reorientation of the dipoles inside the medium occurs and subsequently generates an electrical charge on the crystal faces of the material proportional to the amount of applied mechanical stimulation [2].

**Figure 4:**
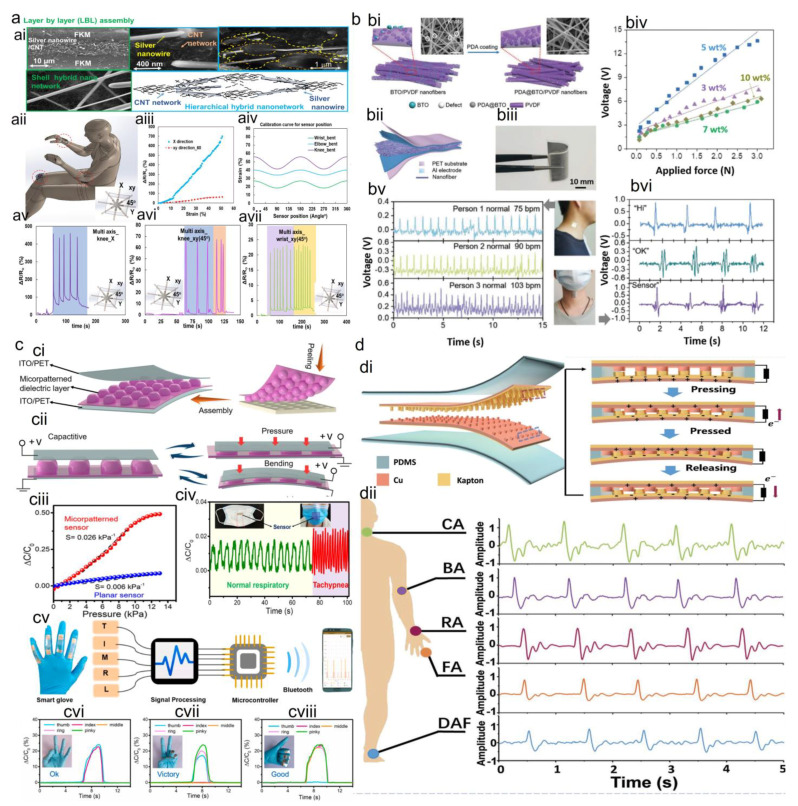
Different sensing mechanisms in wearable sensors for human health monitoring. (**a**) Piezoresistive effect in a multidirectional wearable sensor made of (**ai**) hybrid nanostructures of CNT/AgNW in fluorotelomer FKM for (**aii**) different body joint bending monitoring, including monitoring of knee, wrist and elbow bending; (**aiii**) resultant calibration curve from strain transformation method for different position of sensors with respect to X direction and (**aiv**) resultant calibration curve from strain transformation method for different position of sensors with respect to X direction (or zero degree) and different body joint bending movement. (**av**) Monitoring of knee bending using a multidirectional sensor patch in X directions and, (**avi**) 45° to the X axis, and (**avii**) monitoring of wrist bending using a multidirectional sensor patch at 45° to the X axis. Reproduced with permission from [54]. (**b**) Piezoelectric effect in as-fabricated muscle fiber inspired piezoelectric textile of (**bi**,**bii**) polydopamine (PDA)@ electro-spun barium titanate/polyvinylidene fluoride (BTO/PVDF) nanofibers (MFP) and (**biii**) stretchability of MFP textile by a tweezer, (**biv**) output voltage of the MFP textile with various BTO mass fractions on the external forces, (**bv**) demonstration of pulse waveforms of different testers when wearing the fabricated MFP textile on the same position of their necks, and (**bvi**) dynamic output profile for spontaneous voice recognition when saying different words. Reproduced with permission from [106]. (**c**) Piezo-capacitive effect in the (**ci**) micropatterned surface and capacitive sensor composed of the composites of TPU dielectric layer and ITO/PET electrode and (**cii**) its sensing mechanism under pressure and bending, (**ciii**) demonstration of human physiological monitoring and (**civ**) human respiratory monitoring, (**cv**) the schematic of capacitive sensors applied in sign language interpretation, (**cvi–cviii**) production of Morse codes by pressing the capacitive sensor, and monitoring signal output by smart glove under different hand gestures. Reproduced with permission from [107]. (**d**) Triboelectric effect in a (**di**) flexible self-powered ultrasensitive pulse sensor based on triboelectric active sensor of nanostructured Kapton film and Cu film and (**dii**) demonstration of the signal output pressed on various artery positions. Reproduced with permission from [108].

The triboelectric effect involves friction-induced charge generation and polarization between the two contact materials due to the electrification and electrostatic induction and is not limited to certain materials [1]. This separation of the electrical charges creates a voltage difference. The working mechanism consists of either contact-mode or sliding-mode [104]. Compared to the static signal change in piezoresistive and piezo-capacitive transduction mechanisms, the piezo- and triboelectric devices show an instantaneous response to the applied stress [1]. Using triboelectricity, a new type of self-powered smart sensor was introduced as the triboelectric nanogenerator (TENG) [85], relying on the coupling of contact electrification and electrostatic induction. Different materials used for the fabrication of TENG include paper, fabrics, PDMS, polytetrafluoroethylene, aluminum, polyvinylchloride, and fluorinated ethylene propylene. They can collect energy from a wide range of mechanical motions, such as human motion, vibration, and rotation [85]. By comparison to piezoelectric generators, triboelectric devices could generate higher output power densities and energy conversion efficiencies. Figure 4 shows an example of human health monitoring using different sensing mechanisms including (i) piezoresistive effects for human motion monitoring, (ii) piezo-capacitive for human physiological monitoring and human respiratory monitoring and sign language interpretation by movement of a smart glove for different hand gestures, (iii) piezoelectric for human pulse monitoring and spontaneous voice recognition when saying different words, and (iv) triboelectric for real-time monitoring of pulse signals of the cardiovascular system relative to the diagnosis of coronary heart disease, atrial septal defect, and atrial fibrillation, and arrhythmia (atrial fibrillation). These wearable sensors can also be extensively used to detect various pulse waveforms, an efficient tool in the assessment of cardiovascular diseases. The pressure sensors can be attached to the human wrist to capture the pulse waveforms with typical feature points, such as the starting point, percussion wave, tidal wave, incisura wave, and diastolic wave [105].

##### Sensors’ Performance Characteristics

The performance of strain/pressure sensors based on polymer nanocomposites is assessed by different characteristics, including stretchability, sensitivity, linearity, hysteresis, durability, and similar response time to most other sensors. They depend on various parameters, such as nanomaterials and polymer matrix type, their micro/nanostructures, and the fabrication process.

Stretchability refers to the maximum strain detected by the sensing material with reproducibility. Conventional strain sensors based on metals and semiconductors showed limited stretchability (<5%) due to plastic deformation and brittle failure above the elastic region. Strain sensors based on polymer nanocomposites showed from more than 10% up to 800% stretchability [87]. The stretchability of the strain sensors with 1D nanomaterials, such as CNTs and AgNWs, surpasses the strain sensors with 0D and 2D nanomaterials, such as CBs, NPs, and graphene, which typically are responsive to around 10% strain. The use of 1D materials is a better choice for this application due to their high aspect ratio and effective percolation network [38,89].

Sensitivity or gauge factor is the relative change in the electrical signal, either capacitance (C) or resistance (R), or electrical charge or potential (V or C), versus strain/pressure (slope of the change versus strain/pressure). The sensitivity or gauge factor of the piezoresistive mechanism is the change in the resistance versus the applied strain (ε) or pressure (p) and can be derived by GF = ΔR/(R_o_), where ΔR is the change in the resistance (R − R_o_), and R_o_ is the initial resistance. The gauge factor in a capacitive sensor is GF = ΔC/(C_o_), where ΔC is the change in the capacitance (C − C_o_), and C_o_ is the initial capacitance. The piezoresistive sensitivity in polymer nanocomposite-based sensors is much larger than their piezo-capacitive sensitivities, which are typically less than 1 [38,109]. Conventional metal-based strain sensors showed GF of less than 5, whereas semiconductor-based strain sensors possess much larger GF, up to 200 times more than metals [87]. Sensitivity in piezoelectric materials or triboelectric structures is equal to the difference between input and output voltage or the amount of charge generated versus applied strain/pressure (V/_(_ε or p_)_) or (C//_(_ε or p_)_) [110,111]. These sensors based on polymer nanocomposites can obtain higher GF depending on their sensing mechanism, nanomaterials used, and micro/nanostructures.

Linearity is a factor that indicates how close the output change versus the applied strain/pressure graph line is to a straight line. High linearity makes the calibration process more manageable, but it is not a requirement for calibration [87]. Metal-based sensors and capacitive-type sensors show excellent linearity, but semiconductors have lower linearity because of a significant effect of temperature on their output response [112,113]. Polymer nanocomposite-based sensors offer nonlinear or linear behavior depending on their strain-sensing and pressure-sensing mechanism and morphology [59,63,105,114]. The nonlinearity of the sensors can be compensated by high repeatable and reproducible sensing performance [87].

Hysteresis is the difference between sensor outputs in loading and unloading cycles at the same level of strain/pressure, and this phenomenon affects the dynamic stability of the sensors. The viscoelastic nature of polymers and the interaction between nanomaterial fillers and polymers are the primary reasons that polymer nanocomposites exhibit hysteresis. High levels of hysteresis occur mostly for CNT-based polymer nanocomposites with weak binding [115,116]. However, weak binding is required in metal nanowires, such as AgNWs-based polymer nanocomposites, to avoid high hysteresis [38]. With strong binding, the rigid nature of nanowires causes more friction between nanowires and polymers, leading to buckling and fracturing of nanomaterials upon releasing [117]. The hysteresis phenomenon is inevitable in polymer nanocomposite-based sensors but can be offset using Simulink models, such as Duhem, proposed by Kim et al. [118].

Response time is the time required for the sensor to reach steady state [119]. Recovery time is the time required for the sensor to return to its original state upon unloading in a dynamic test. The response times in most polymer nanocomposite-based sensors are in the range of milliseconds; for instance, a response time of less than 22 ms was reported for AuNWs/silicon-based elastomers [100], 200 ms for AgNWs/PDMS [101] and 332 ms for CNTs/Ecoflex stretchable strain sensors [45]. The recovery time for polymer nanocomposite-based sensors is longer due to the viscoelastic effect of polymer nanocomposite and the friction between fillers and polymers [119].

The reliability of a sensor refers to its ability to function consistently without degradation. Zero-point drift, environmental conditions, and low cyclic stability can cause degradation. Cyclic durability is the long-term stability of a sensor in terms of electrical functionality and mechanical integrity in several stretching–releasing cycles. Any plastic deformation of the polymer matrix and buckling of sensing nanomaterials can cause dynamic instability [38].

**Figure 5:**
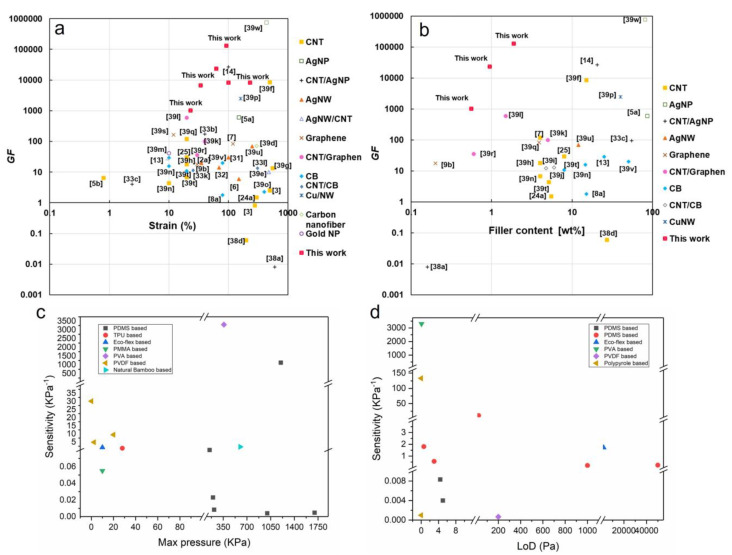
Summary of the results from various polymer-based stretchable strain sensors obtained from the literature. (**a**) Maximum GF achieved for different composites including metal-based and carbon-based single/hybrid fillers corresponding to the reported max strain. (**b**) Maximum GF achieved versus filler concentrations. Reproduced with permission from [73]. (**c**) Summary of the literature for various flexible pressure sensors based on their polymeric based dielectric layers for sensitivity versus maximum pressure detection and (**d**) sensitivity versus minimum pressure as of limit of detection (LoD). The data are plotted from Table references [121,122].

Regarding strain sensors, there is a trade-off relationship between “high sensitivity (GF > 50)” and “high conductivity (>1 S.cm^−1^) and stretchability (>100%)” in most of the recently developed strain sensors [38]. In addition, most sensors based on polymer nanocomposites often used high filler concentrations (>10wt%) [73]. Figure 5a,b summarize the current literature on strain sensors’ performance for sensitivity, stretchability, and filler concentrations. It can be seen that only a few studies used low filler concentrations (<2 wt%). Therefore, considerable structural deformations upon stretching require a highly sensitive response. In contrast, the sensor morphology and conductivity change should be mild under large stretching in order to have a high stretchable strain sensor. Both features are hard to achieve through simple thin film sensor structures. To circumvent this difficulty, a few methods were shown to be useful, including modifying nanostructures inside the polymer [54,73] or surface strain delocalization in the conductive film on the polymer substrates [120].

Figure 5c,d compare different pressure sensors in the literature that used different polymer nanocomposites for their dielectric layers [121,122]. For pressure sensors, it is challenging to obtain high sensitivity (GF > 50 kPa^−1^), and wide pressure ranges (LoD < 10 Pa to Max pressure > 100 kPa).

#### 2.1.2. Chemical/Biosensors

Biosensors are sensing devices that incorporate a biological recognition element into the sensor operation (for example, aptamer, enzyme, antibody, cell receptor, or organelle). A typical biosensor contains two basic functional units: a ‘bioreceptor’ (for example, enzyme, antibody or DNA) responsible for selective recognition of the target analyte, and a physicochemical transducer (for example, electrochemical, optical, or mechanical) that translates this biorecognition event into an electrical signal. Aptamers are single-stranded oligonucleotides that can bind to the target molecule, similarly to antibodies but with enhanced specificity [123]. Enzyme-based biosensors are typically more selective and specific than other sensor materials and have an increased response time due to shorter diffusion paths [124]. Antibodies are protective proteins released by the immune system in the presence of an antigen [125]. They perform chemical analysis of different biofluids, including tears, saliva, urine, blood, interstitial fluid (ISF), and sweat.

Among them, sweat, tears, saliva, and ISF can be samples in a non-invasive manner and offer minimal risk of harm or infection to the skin [126]. Frequent techniques for biomarker analysis are immunoassays, including lateral flow assays, enzyme-linked immunosorbent assay, colorimetric detection, surface plasmon resonance, and electrochemical response. The technology of immunoassay detection is based on the target analyte’s interaction with its matching antibody. The amount of antigen that binds with antibodies in the sample is used to measure the concentration of the antigen present. In immunoassays, transduction can be visual/colorimetric, fluorescence, and chemiluminescence based [125]. Surface Plasma Resonance is a label-free detection method that uses refractive index changes to determine target analyte levels. The refractive index changes when a biorecognition element (antibodies, RNA aptamers, AuNPs) binds with the target analytes. Colorimetric includes quantitative measurements of color intensity upon biomarkers’ reaction with the assay and analysis of digital images collected from the microfluidic structures [127]. Electrochemical biosensors typically consist of a bioreceptor and a transducer that converts electrochemical data into electrical signals to quantify biomarker levels [126]. The receptors in the sensors can be enzymes, antibodies, aptamers, or DNA. Electrochemical detection is highly sensitive and can detect biomarkers in the femtomolar ranges [125].

#### 2.1.3. Wearable Sweat Biosensors

**Figure 6:**
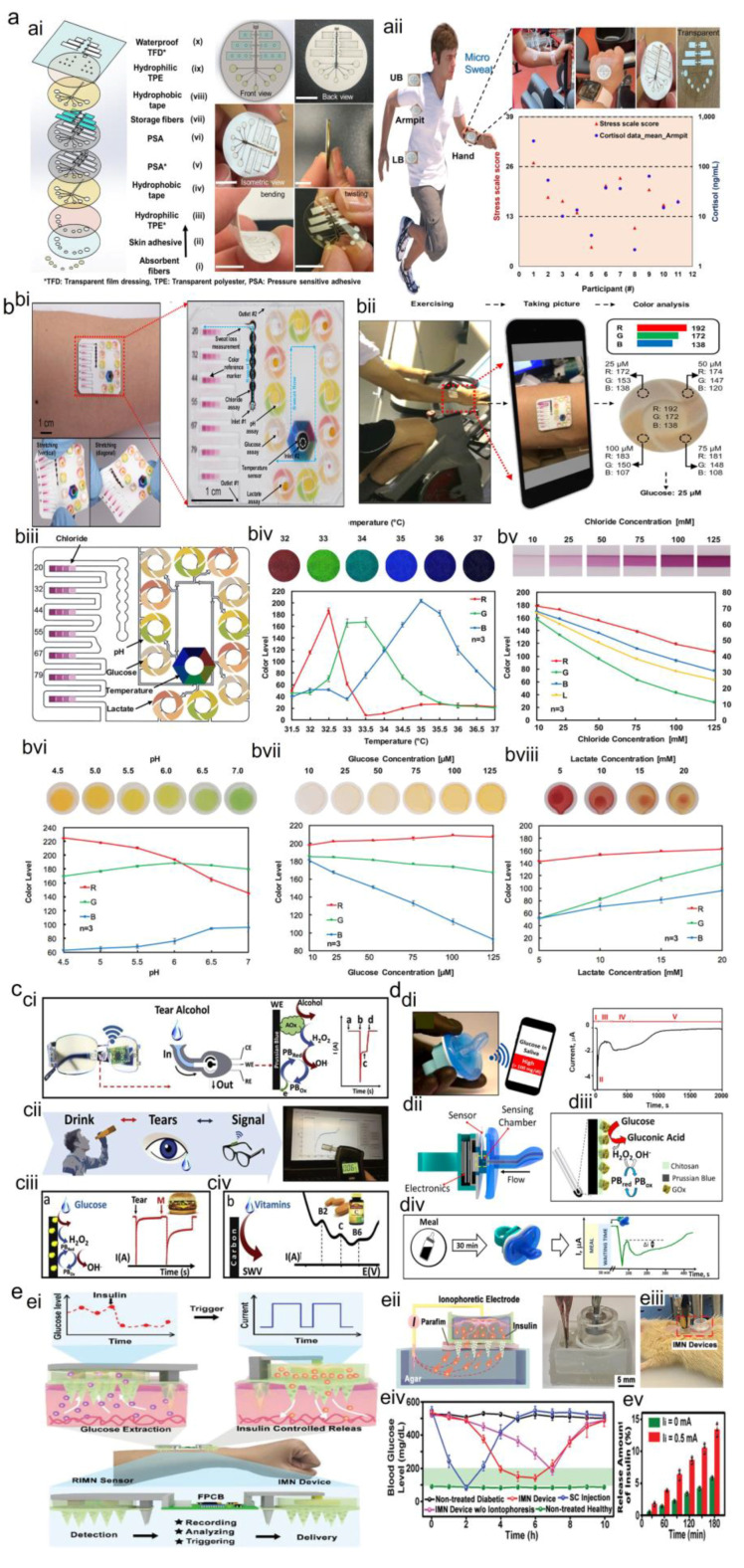
Wearable devices for biofluid collection and sensing. (**a**) Wearable MicroSweat for sweat collection and monitoring of sweat cortisol with (**ai**) demonstration of its different layers and flexibility and (**aii**) positioning of MicroSweat in different body locations, measuring the correlation between sweat cortisol and stress levels in humans, Reproduced with permission from [129]. (**b**) Wearable microfluidic devices for colorimetric analysis of sweat on (**bi**) the skin and under mechanical deformation with bending and twisting, and (**bii**) procedure for sweat collection and colorimetric analysis for (**biii**–**bviii**) monitoring of chloride, glucose, pH, and lactate in sweat and body temperature. Reproduced with permission from [130]. (**c**) Wearable eyeglasses-based fluidic device for (**ci**–**ciii**) enzymatic detection of alcohol and glucose in tears and (**civ**) square-wave voltammetry of tear vitamins, such as vitamin B_2_, B_6_ and C. Reproduced with permission from [152]. (**d**) Wearable glucose pacifier for electrochemical sensing of glucose in the saliva of newborns, (**di**,**dii**) wireless pacifier biosensor working-principle for on-body saliva monitoring, (**diii**) the glucose enzymatic biosensing approach on the PB electrode, and (**div**) the procedure used for on body glucose sensing experiments. Reproduced with permission from [158]. (**e**) A fully integrated wearable closed-loop system based on (**ei**) a porous microneedle (MN) platform coupled with iontophoresis for subcutaneous substance exchange for both glucose extraction and (**eii**) in-vitro insulin delivery, (eiii) photographs of the experimental setup of MN device and application of MN device on anesthetized rats, (**eiv**) facilitating diabetes therapies via iontophoretic MN device, non-iontophoretic MN device, and subcutaneous injection of insulin. (**ev**) Measurement of insulin released from the MN device for 180 min in 3 measurements. Reproduced with permission from [164].

Sweat is considered an ideal biofluid for detecting biomolecules in wearable Point of Use devices because it is much easier to collect for testing than other biofluids, such as blood, which require invasive methods [128]. With more than 100 glands/cm^2^, sweat glands are distributed across the body. Sweat generally contains metabolites (lactate, glucose, urea, and cortisol), electrolytes (ammonium, chloride, and sodium), trace elements (e.g., zinc), and other large molecules (proteins, nucleic acids, and cytokines) [126]. For non-invasive monitoring of physiological health status (e.g., hydration or physical stress) and disease diagnosis and control, in situ sweat analysis is of significant importance [127]. Several studies have found different microfluidic platforms for sweat collection [129,130,131] and biomarker detection. For instance, the Rogers group found that wearable colorimetric/fluorometric microfluidics were a suitable platform for various sweat biosensing, such as sweat rate [132], electrolytes [133], pH [134], vitamin C [135], lactate [136], glucose [136], creatinine [132] and, ammonia and ethanol [137]. Javey group, Gao group and Wang group also focused on electrochemical biosensing of sweat using various wearable sweat patches for sweat rate [30], L-Dopa [138,139], and C-reactive protein [140], cortisol [141,142], ketone [143], glucose [144], and therapeutic drugs [145]. Figure 6a,b show examples of wearable sweat collection and sensing devices for sweat cortisol, alcohol, and ammonia monitoring.

#### 2.1.4. Wearable Tear Biosensors

Tears contain protein, peptides, lipids, metabolites, and electrolytes [126]. They are an attractive biofluid for non-invasive monitoring of various metabolites and physiological parameters due to close tear–blood concentration correlations [146]. Wearable tear biosensors have been used for continuous glucose monitoring since 2007 [147,148,149], and later for lactate sensing since 2012 [150]. In addition, tears were used for monitoring human eye intraocular pressure as an indicator for glaucoma, which can cause blindness, using soft contact lenses for the wireless detection of both glucose and intraocular pressure [151]. In that work, the glucose sensor channels consisted of graphene electrodes on a field effect transistor (FET) pattern. Most wearable contact lenses also use electrochemical sensors for tear biomarker detection. In one of the most recent works, Sempionatto et al. [152] used a non-invasive wearable tear biosensor system mounted on eyeglasses for real-time monitoring of different analytes, including alcohol, vitamins such as vitamin B_2_ and B_6_, and glucose in tears (Figure 6c). It could collect stimulated tears on the external miniaturized flow detector mounted on the eyeglasses pad. It consisted of electrochemical sensors on the (polyethylene terephthalate) PET substrate, including a carbon electrode for glucose sensing, a mixture of Carbon Prussian Blue (PB) and glucose oxidase for glucose, PB and alcohol oxidase for alcohol sensing, and a wireless electronic backbone outside the eye area.

Optical sensors were also employed for glucose monitoring of tears using a photonic microstructure on the surface of hydrogel sensor networks and contact lenses [153]. Their mechanism was based on the volumetric changes of the hydrogel in response to the variation in glucose concentration and, thus, changing the diffraction angle and the zero/first-order interspacing for the diffracted monochromatic light in transmission mode. They achieved fast and facile preparation, swift response at low glucose concentrations, and simple readouts within physiological conditions. Despite some efforts, several challenges remain, such as difficulty in sampling tears, small sample volumes, issues with fast evaporation, dependence of tear composition on emotional or mechanical stimulation, and reliable operation of fully integrated wireless sensing platforms [126,152].

#### 2.1.5. Wearable Saliva Biosensors

Saliva is an oral biofluid that can originate either from salivary glands, microorganisms, or blood diffusion through transcellular and paracellular pathways [154]. It contains water, electrolytes, cells, enzymes, hormones, metabolites, and antimicrobial agents [146]. Saliva is one of the ideal biofluids for detecting body nutrition upon taking foods or supplements, since it shows the quickest response, e.g., ketone bodies or vitamin C and vitamin D [155] can be detected in saliva within 30 min. Wearable saliva biosensors can have different configurations, such as a wireless mouthguard biosensor used by Kim et al. [156] to detect uric acid to prevent diseases such as hyperuricemia, gout, and Lesch–Nyhan syndrome. The mouthguard biosensor is comprised of printing technology on a flexible PET substrate, PB transducer, uricase enzyme, and electropolymerized o-phenylenediamine. Another structure can be tooth enamel incorporated with printed wireless graphene nano-sensors on biomaterials via silk bio-resorption for bacteria detection in saliva [157]. These bio-transferrable sensors exhibited high sensitivity and selectivity and promised pathogenic threats. In another work [158], a fully integrated wearable pacifier-based platform was used for glucose monitoring in newborns’ saliva. They demonstrated on-body performance for glucose sensing in saliva for real-time tracking of type I diabetes in saliva (Figure 6d).

Although wearable saliva biosensors have the advantages of rapid collection, large analyte volume, continuous flow, and relatively clean samples for reliable measurements, they still suffer from bulkiness and discomfort in practical applications [146]. Therefore, careful consideration is required to design a platform for long-term and clinical use.

#### 2.1.6. Wearable ISF Biosensor

ISF is a biofluid surrounded by the cells inside the body. It fills the extracellular space between tissues and transports various chemical species to the cells, such as metabolites, proteins, nitrogenous waste, chemical messengers, and lipids. In addition, various substances are exchanged with blood capillaries by exchanging oxygen and carbon dioxide for cellular respiration. While ISF has the closest composition to the blood, with biomarker concentrations and temporal profiles similar to those in blood [159], some proteins can only be found in ISF, making it a powerful and unique candidate for wearable biosensor devices.

Various devices have been used for ISF extraction and monitoring, including microneedles [160] with sensors installed on the needles or attached externally, reverse iontophoresis (RI), and electrochemical sensing [161,162], and implantable devices. ISF can be used for continuous monitoring of biomarkers, including in a recent work by Teymourian et al. [163] for continuous glucose monitoring (CGM) and continuous ketone monitoring using a microneedle sensor array. The β-hydroxybutyrate microneedle sensor was coupled with an oxidase-based glucose microneedle sensor on the same array platform for real-time monitoring of these diabetes markers. Moreover, they detected lactate in ISF using a similar platform and lactate oxidase enzyme. In addition to CGM, simultaneous drug delivery can occur. Li et al. [164] employed mesoporous microneedles patches for both glucose extraction and insulin delivery (Figure 6e). The simultaneous microneedle and RI could increase glucose extraction or insulin delivery. This fully integrated wearable closed-loop system (IWCS) could accurately track glucose concentration via electrochemical sensors and accordingly deliver insulin. This IWCS platform could regulate blood glucose levels in a diabetic rat. Similarly, another research study [165] used phenylboronic acid units and ISF glucose to trigger the insulin release based on the weakening of the electrostatic interactions between the insulin and microneedle matrix in a mice model. Several other studies also focused on CGM using microneedle-based sensors [166,167,168].

Hair follicles are another passageway for ISF extraction using the RI method. So far, a transdermal patch consisting of graphene microarrays has been developed to monitor ISF glucose through follicles [169]. They employed hyaluronic acid to increase osmotic pressure and drive more glucose toward the electrode.

Although current wearable devices based on RI and/or microneedles showed promising results for ISF extraction, sensing, and drug delivery, several issues remain unsolved.

The limitations include possible skin irritation due to the passage of electric current or needle passage, cross-contamination from sweat, and sampling of extremely low fluid volumes after long operating periods in RI methods [170]. Although combining microneedles and RI has been offered as a solution to this issue, finding user-friendly and more accurate methods can be a breakthrough in this area.

#### 2.1.7. Optical Sensors

Optical transduction in wearable sensors involves the conversion of physical or chemical parameters into measurable optical signals. High sensitivity, non-invasiveness, and immunity to electromagnetic interference make them suitable for a wide range of wearable applications [1,171]. Optical sensing methods can be divided into label-based and label-free methods. Standard label-free techniques include optical fiber, ring resonator, interferometer, optical waveguide, photonic crystals, fluorescence/luminescence, surface plasmon resonance, absorbance, reflectance, and refractometry [172]. Another optical sensing technique is photoplethysmography for vital sign monitoring, which enables continuous and non-invasive measurements of heart rate, blood oxygen saturation, and even blood pressure [20]. Label-based optical techniques are colorimetric or fluorometric methods [172] which have demonstrated great promise for monitoring of metabolites, glucose, and other biomarkers in sweat, tears, or interstitial fluid, providing valuable insights for disease management and preventive healthcare [173,174].

Optical sensing platforms often employ nanomaterials, including silver and gold nanoparticles [175]. Recent studies have focused on developing novel nanomaterials for optical transduction in wearables to improve sensitivity and selectivity, such as quantum dots, carbon dots, and plasmonic nanoparticles with exceptional optical properties [176,177,178]. Integration of these materials into flexible substrates and microfluidic devices has facilitated the development of wearable devices that are lightweight, conformable, and comfortable for long-term use [172].

Compared to electrochemical methods, optical biosensing can provide meaningful continuous monitoring with minimum fouling or signal drift depending on the materials and geometries [179]. In electrochemical methods, biofouling occurs with electrodes, leading to a decreased sensitivity for a specific analyte. Optical sensors can provide continuous analysis. In addition, some optical transducer systems with spectral analysis or imaging for sample analysis have no direct contact with the sample [179]. Despite these advantages, optical transduction mechanisms in wearable biosensors face certain challenges. The significant challenges are interference from ambient light and motion artifacts, which affect measurement accuracy and require advanced signal processing algorithms and motion compensation techniques [180]. Additionally, the calibration and stability of wearable optical sensors remain critical issues, demanding ongoing research and standardization efforts.

#### 2.1.8. Electrophysiological Sensors

Electrophysiological sensors can monitor subtle changes in electrical potential and impedance related to the movement of muscles in human organs. Any muscle movement or contraction creates electrical depolarization signals that can be transferred to the skin [1]. Electrical transduction involves the conversion of physical signals into measurable electrical signals. This mechanism offers advantages such as high sensitivity, ease of integration, and compatibility with electronic devices such as mini biopotentiostats. Wearable electrophysiological sensors covert various electrophysiological signals, including those obtained in electrocardiography (ECG), electroencephalography (EEG), electromyography (EMG), and electrooculography (EOG).

In this regard, wearable ECG sensors continuously monitor heart activity and facilitate early detection of arrhythmias and cardiovascular abnormalities [181]. Wearable EMG and EOG sensors measure muscle activity and have advanced fields, such as rehabilitation, robotics, and sports medicine [173,182]. Wearable EEG sensors can monitor brain activities and are essential in tracking encephalopathies, such as stroke, brain tumor, schizophrenia, and epilepsy [1]. Furthermore, bioimpedance-based wearable sensors offer non-invasive monitoring of physiological parameters, like hydration, body composition, and respiratory function [183,184].

In these electrical transduction mechanisms, the need to decrease sensor size, enhance sensitivity, minimize power consumption, and improve signal-to-noise ratios to ensure precise and dependable measurements is challenging. Moreover, ensuring the compatibility of wearable ECG and EMG sensors with diverse body types and anatomical variations presents significant design and engineering obstacles.

### 2.2. Current Limitations of Wearable Sensors

The most significant limitations of wearable sensor technology for early detection of health are sensitivity, accuracy, and technology validation. The first-generation wearable sensors have shown potential in monitoring physical activity and other health metrics, particularly in chronic disease management. The second-generation wearables must improve accuracy, battery life, data security, user adoption, and standardization [14,16]. The need to recharge electrical devices frequently can limit their effectiveness in monitoring health status over an extended period, which is particularly problematic for those with chronic health conditions. The lack of standardization with gold standard methods in data analysis makes interpretation difficult and unreliable. This can make it challenging to compare data across different devices and make informed decisions about patient care. By overcoming these limitations, wearable technology can become a more effective tool for improving health outcomes and reducing healthcare costs.

## 3. Integration of AI in Wearable Health Technology

### 3.1. Overview of AI Techniques Applied to Wearable Sensors

#### 3.1.1. Machine Learning 

ML algorithms are valuable in analyzing the extensive data gathered by wearable devices, empowering healthcare providers to identify patterns, predict outcomes, and make suitable decisions regarding patient care. They are classified into supervised and unsupervised learning [185]. One of the most notable advantages of these algorithms is their ability to discern subtle changes in health metrics, which could indicate early signs of disease or other health issues. Over the past years, there has been a substantial surge in research focused on applying ML algorithms to healthcare wearable technology [6,12].

For instance, these algorithms were employed to predict the onset of chronic diseases, such as diabetes, cardiovascular disease, and mental health issues, by measuring heart rate and blood glucose meters [4,186]. By detecting patterns in the data, healthcare providers can intervene early and prevent the progression of these conditions. Moreover, ML algorithms have been instrumental in monitoring and managing chronic diseases, like Parkinson’s and multiple sclerosis [187,188]. They analyze data from wearable devices, such as accelerometers and gyroscopes, to monitor motor symptoms and allow healthcare providers to adjust medication dosages [189,190]. Additionally, these algorithms have been utilized to monitor mental health conditions like depression and anxiety through wearable devices like smartwatches and fitness trackers.

Two neural networks were developed in the ML algorithms. (i) The first is an artificial perception and transmission nerve (APT) nerve, which emulates the functions of biological sensory neurons. The APT nerve can detect mechanical stimulation, discern the location of the stimulation, and transmit mechanosensitive signals. The APT nerve was employed for playing music, controlling positioning in a two-dimensional plane, and handling rotation [191]. APT holds promise for applications in socially intelligent robotics and smart prosthetics. (ii) Human activity recognition using deep multi-task learning (AROMA), a convolutional neural network, identifies basic activities without relying on human-made rules. For more complex activities, AROMA utilizes a “long-term memory network” to understand the order and context of actions, demonstrating impressive recognition performance comparable to other methods in this domain [192]. For instance, bidirectional long short-term memory (LSTM) technology could categorize patients’ health conditions based on their data related to diabetes, blood pressure, mental health, and drug reviews. It was built using a tool called Protégé Web Ontology Language with Java to correctly classify healthcare data and predict drug side effects and abnormal conditions in patients [193].

A significant constraint of applying ML algorithms to wearable technology is the lack of standardization in data collection and analysis. Comparing data across a wide range of wearable devices becomes challenging, compromising the efficacy of ML algorithms in classifying health conditions [20,194]. To address this challenge and improve accuracy, cloud computing and a big data analytics engine were developed to store and analyze healthcare data efficiently.

#### 3.1.2. Deep Learning

Deep learning (DL) algorithms are a subset of ML algorithms employing multi-layered artificial neural networks to identify intricate patterns and find relationships within massive datasets [194]. Various DL implementation frameworks for high-performance computing platforms are open source: TensorFlow, Theano, Caffe, Pylearn2, Torch, Cognitive network toolkit, and Lasagna [194]. These frameworks have paved the way for automatic feature learning in human activity recognition, benefiting from increased computational power and access to large datasets from wearable sensors, Internet of Things (IoT), and crowdsourcing.

DL algorithms have been proven effective in the early detection and preventing of chronic diseases, like diabetes and cardiovascular disease [17,195]. In addition, they can analyze wearable ECG data, enabling the detection of subtle changes in heart rhythms that could indicate cardiac abnormalities [196]. Additionally, DL algorithms have been used to detect early signs of sleep apnea, a common sleep disorder with potentially severe health consequences [197]. Similarly, they have facilitated the identification of symptoms of epilepsy through wearable data, enabling early intervention and treatment [198]. Researchers also employed an artificial neural network (ANN) to estimate knee joint forces (KJF) during sports movements. The ANN accurately predicted vertical and anterior-posterior KJF values for most movements, showcasing the potential of wearable sensors combined with ANN for estimating joint reactions in sports [199].

There are several limitations to the application of DL algorithms to wearable technology. These algorithms require substantial datasets for practical training, necessitating standardized data collection and analysis procedures to ensure high-quality data. Addressing these limitations will be crucial for unlocking the full potential of DL in healthcare wearable technology.

### 3.2. AI-Based Data Processing for Wearable Sensor Data

#### Signal Processing and Noise Reduction

AI has emerged as a crucial tool for signal processing and noise reduction in wearable devices by identifying patterns in sensor data, detecting anomalies, and distinguishing between signal and noise [200,201]. AI algorithms effectively enhance the quality of the signals while filtering out unwanted noise.

In recent years, an interest has been growing in leveraging AI for human activity recognition. Traditional algorithms struggled to achieve high accuracy due to the complexity and variability of human activities and high signal-to-noise ratio data. To eliminate noise, a novel approach, hierarchical DL based on LSTM (H-LSTM), was used [193]. The H-LSTM method is then applied to classify different activities. The approach was tested on three public databases from the University of California, Irvine, ML repository to assess its performance. The results demonstrated that H-LSTM outperformed other DL methods, achieving an impressive 99.15% accuracy in recognizing human activities. This novel approach shows potential in advancing the accuracy and effectiveness of noise-free wearable sensor data using AI.

Moreover, AI plays a crucial role in improving the data normalization and transformation of wearable technology [14,202]. By converting data into a standard format, AI algorithms can ensure that the data is easier to analyze and compare. They can detect anomalies in sensor data, such as outliers or missing data, and transform them to remove them, ensuring that the data is accurate and reliable. AI can also personalize individual users’ data normalization and transformation processes by learning their behavior patterns [202]. This personalization is significant in wearable devices for human health monitoring.

AI algorithms can be optimized for real-time sensor data processing, making them ideal when quick response times are critical, such as getting feedback from wearable glucose sensors for diabetic disease. This capability is critical for emergency response scenarios or sports training, where quick response times can save lives. For instance, researchers designed a smart wristband equipped with a non-invasive sweat alcohol sensor and an IoT-based alarm system known as “Drunk Mate.” This wristband could detect alcohol levels in real-time and send alerts to the user’s smartphone when their blood alcohol concentration reaches an illegal limit [174]. With high accuracy and specificity to ethanol, this device presents a promising and cost-effective solution for non-invasive and real-time alcohol measurement and monitoring.

### 3.3. AI Applications for Real-Time and Personalized Health Monitoring

#### 3.3.1. Disease Prediction and Diagnosis

Compared to the previously time-consuming and expensive lab-based diagnostic tools for disease diagnostics, including precision screening of prostate cancer [203], breast cancer [204], lung cancer [205], brain lesion [206], coronary artery disease [207], diabetes [208], esophageal adenocarcinoma [209], COVID-19 [210], heart failure [211], skin cancers [212], AI-based techniques are facilitating real-time monitoring capabilities and personalized health monitoring. Therefore, with more accurate data, disease treatment can be carried out on an individual basis. ML would help AI training concerning the big data resources from past datasets of millions of patients, including medical records, omics, medical imaging, and wearable diagnostics, in order to recognize patterns of disease conditions and progression and make clinical decisions faster [16].

The importance of AI tools for wearable biosensors has recently been shown to improve health monitoring, especially for chronic disease [213]. AI-based wearable biosensors were used for cancer detection, such as using a dual-gate FET to detect prostate cancer in urinary samples with multiple biomarkers. With the help of random forest (RF) and neural network algorithms, the screening performance was improved with more than 99% accuracy using surface-enhanced Raman spectroscopy signals of 76 urine specimens for multiple biomarkers’ detection, including ANXA3, ENG, PSMA, and ERG [203]. Another example of using wearable AI-based biosensors is the detection of volatile organic compounds (VOCs) using ANN model-assisted Si-nanowire FETs in breath prints from lung cancer, gastric cancer, and asthma and chronic obstructive pulmonary disease patients, and early diagnostic stages of cancer [214,215]. Here, using ANN would improve the selectivity and sensitivity of sensors in mixtures containing counteracting compounds with similar chemical structures and physical properties [216]. In addition, multiple numbers of VOCs and their binary and ternary combinations can be recognized with this method. AI technology can also be designed with wearable devices to track motion activities, pulse rates, and heart rates for cardiovascular diseases. The speed and accuracy levels of the devices could be adjusted to give the perfect measurements of physical reaction, heart rates, and energy levels [217]. Recently, AI techniques, with the help of a deep learning model including Recurrent neural networks (RNN) and convolutional neural network (CNN) algorithms, could predict and diagnose coronavirus using an Oura smart ring rapidly within 24h [218].

Combining the IoT, cloud computing and AI have helped researchers with the diagnosis of diabetes and heart disease [208]. In this regard, wearable sensors were employed as IoT devices to perform the data acquisition, and AI techniques were used to process this data for disease diagnosis. A Crow search algorithm was used based on an LSTM model combined with isolation forest algorithms to improve the diagnostic accuracy. This combined algorithm obtained maximum accuracies of 97.3% and 96.2% for diabetes and heart disease diagnosis, respectively, which is very promising for healthcare wearables.

#### 3.3.2. Treatment and Feedback

AI-based wearable devices can also be used to manage diseases by in situ therapy, thus helping physicians with faster decision-making and effective treatment. In this regard, several studies focused on using wearable microneedles to deliver drugs to diabetic patients based on feedback provided by continuous and real-time monitoring of glucose levels in different biofluids. Here, the AI techniques can determine the level of drugs, depending on the disease stage, to avoid overtreatment or mistreatment. In this regard, Keum et al. [219] used a smart contact lens with an electrochemical biosensor to continuously monitor glucose levels in tears and, thus, perform diabetic diagnosis and diabetic retinopathy therapy. Lee et al. [220] proposed a combined system of sensing and treatment using sweat-control components, sensing components (humidity, glucose, pH, and tremor sensors), and therapeutic components (microneedles, a heater, and a temperature sensor). The drug delivery is activated once the microneedle coating layer of the phase-change material is heated up to the phase transition temperature. They performed in vitro tests and therapy on diabetic mice, with feedback treatment when hyperglycemia was detected based on the results of glucose level monitoring.

Therapy efficiency can be enhanced by real-time feedback systems from therapist, user, and cloud systems. For instance, in CGM systems, besides feedback at the biomarker level, another form of feedback can be nutrient levels by employing wearable sensors systems and advanced AI-powered data-fusion algorithms to manage nutritional imbalance toward maintaining desirable nutrient levels [221]. Wearable biosensors could determine the levels of nutrients, such as glucose, salts, and vitamins in the body. AI can access databases with calories and nutritional information for the captured food images by an external sensor [221]. This would revolutionize the user’s behavior and let diabetes patients have access to faster dietary decision-making processes and, thereby, follow a healthier diet and lifestyle.

## 4. Case Studies: AI-Enabled Wearable Sensors for Health Monitoring

### 4.1. Physical Sensors

#### 4.1.1. Activity Trackers and Smartwatches

One of the challenges in wearable physical sensors for human activity tracking is distinguishing between different signals’ amplitude and frequency among different ranges of motion. AI and machine learning techniques can help wearable physical sensors accurately classify similar or slight movement responses and classify regular and irregular signals. Therefore, the next generation of wearable sensors for human activity tracking systems uses smart watches for wireless communications and combines machine learning for daily healthcare systems. Lin et al. [222] employed an ultralight sensing material based on radial anisotropic porous silver fiber to collect four types of throat motion signals, including two speaking signals (“Goodbye” and “Hello”), swallowing, and coughing. They employed machine learning models, including XGBoost, k-nearest neighbor (KNN), and support vector machine (SVM), to effectively classify the four types of throat motions and noise signals above. The AI helped sensors with throat state identification at an accuracy above 85% and the early diagnosis of viral throat illness. In another attempt, Kim et al. [223] fabricated AI-enabled piezoelectric yarns made of BTO nanoparticle-enhanced P(VDF-TrFE) fibers for tracking everyday human activities, such as jumping, running, stair climbing, standing still (standing), working out on a stepper, tiptoeing, and walking, along with the corresponding snapshots for each motion activity. Their sensor could detect different patterns depending on the type of motion, but the signal obtained from the same activity for each measurement slightly varied. Therefore, to better identify human activities, a CNN with a multi-kernel was considered to efficiently classify the motion signals captured from the body through piezo-yarns, yielding an accuracy of 99.6%.

Surface electromyography (sEMG) is a bioelectrical signal emitted from neuromuscular activity [224] that can be used for speech tracking by attaching sEMG electrodes to the surface of the human skeletal muscle. AI and machine-learning techniques can help decode sEMG signals, enhancing the reliability of information recognition. Liu et al. [225] fabricated an epidermal sEMG tattoo-like patch that could be laminated on the jaw and face and monitored the sEMG from a patient who had lost their voice. The action instructions from silent speech recognition to control an intelligent car, emotion instructions to control a Bluetooth speaker, and virtual interaction in real time could be recognized with high accuracy using wavelet decomposition and machine-learning algorithms.

#### 4.1.2. Gait Analysis and Fall Detection

Parkinson’s is the second most common progressive neurodegenerative disease, which can be classified into tremor-dominant and postural instability and gait difficulty forms. Gait disorders are associated with losing independence and an increased risk of falls. Recent (AI)-based wearables are helping with early diagnosis, diagnostic accuracy, long-term monitoring of Parkinson’s’ disease (PD), and better management of therapeutic strategies. Ilesan et al. [226] offered a wearable gait assessment system with pressure sensors on a physiograph and convolutional neural network (CNN). This enabled bilateral tracking of the foot biomechanics by means of plantar pressure distribution and lower-limb EMG in correlation with upper limb balance.

In another work [227], using wearable capacitive sensors integrated with textile socks, the walking gait during daily activities was monitored. An adaptive neuro-fuzzy network (ANFIS) was employed to accelerate the convergence of intelligent textiles and machine learning algorithms for the assessment of human gait phases, including heel-strike (HS), heel-off (HO), toe-off (TO), and mid-swing (MS). The proposed ANSFIS model reached a mean performance accuracy of 93% (for the TO phase), 94% (for the MS phase), 96% (for the HO phase), and ≈100% (for the HS phase). One of the significant challenges in fall detection in PD is correlating data from walking gait analysis with body health status and facilitating disease prognosis and management [228].

### 4.2. Chemical Sensors

#### Biofluids Monitoring

Integrating biosensors in wearables goes beyond data collection; it extends to intelligent analysis and actionable feedback. With cutting-edge AI and ML algorithms, these devices provide users with personalized health recommendations, early detection of potential issues, and even life-saving interventions [177,183,184,229,230,231].

**Figure 7:**
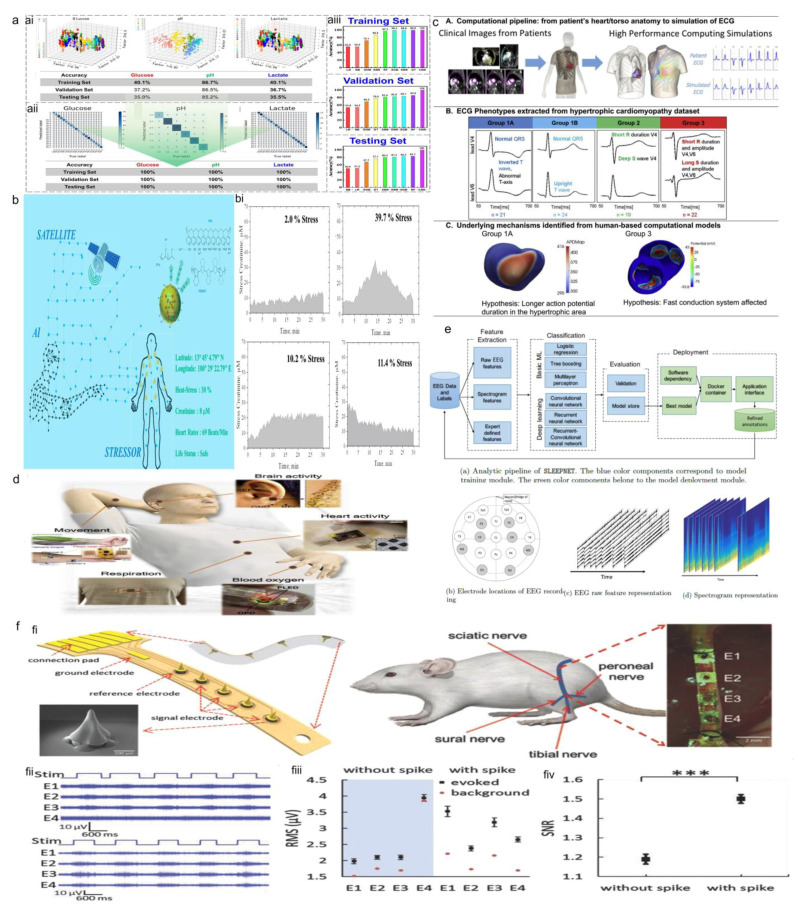
(**a**) ML and DL models of various sweat biomarkers, (**ai**) three-dimensional LDA score plot and classification accuracy for glucose, pH and lactate, (**aii**) confusion matrixes and classification accuracies of CNN prediction for glucose, pH and lactate, and (**aiii**) comparison of prediction accuracies by different classification models including ANN, XGBoost, DT, KNN, LR, NB, RF, SVM, and CNN on the training set, validation set, and testing set. Reproduced with permission from [233]. (**b**) Remote AI-based telemedicine sensing and monitoring platform for stressor detection using the satellite-tracking mobile system for (**bi**) monitoring the activities of exercising outdoors, planting outdoors, reading indoors, and exercising in a hot confined room during half-hour periods, Reproduced with permission from [235]. (**c**) Computational modelling and simulation for understanding the ML outcomes for Panel A: the simulation of the ECG through biophysically detailed computational models, Panel B: ECG phenotypes extracted from clustering techniques, and Panel C: the mechanisms identified by computer models for the ECG phenotypes. Reproduced with permission from [181]. (**d**) A newly proposed PSG setup with recently developed wearable sensors and electronics systems for sleep monitoring at home. They can measure brain activity, heart activity, blood oxygen saturation, respiration, and movement, Reproduced with permission from [241]. (**e**) An architecture of SLEEPNET consisting of a training module and a deployment module for an accurate annotation algorithm that can take the multi-channel EEG data as input and automatically output a sequence of sleep stages, with one stage label assigned to each 30 s epoch, Reproduced with permission from [244]. (**f**) Design and fabrication of the SUN interface, (**fi**) schematic of the SUN interface, with diagram of the SUN interface implanted on the rats’ sciatic nerve, (**fii**) implantation and measurement of impedances of both and ENG recordings responding to limb flexion, and (**fiii**,**fiv**) final RMS comparison and SNR comparison (Statistical significance for *** was defined for *p* < 0.001). Reproduced with permission from [247].

AI technology has shown great potential for improving sensitivity and data analysis in biosensor platforms. For instance, sweat biosensors offered several types of potential, such as colorimetric sensing of different sweat biomarkers like glucose and lactate [232], and pH. Using a DL assisted image analysis, Liu et al. [233] tried to address the inevitable drawbacks of using colorimetric assays, such as non-uniform color development, in colorimetric spotting, color leaching, and chemical diffusion in microfluid devices. Figure 7a shows glucose, pH, and lactate in human sweat could be easily and accurately classified and quantified by the CNN and DL algorithm and the testing results of actual sweat via the DL-assisted colorimetric approach matched 91.0−99.7% with the laboratory measurements [233]. In addition, AI is a great tool to predict human hydration levels after exercising using physiological responses and sweat biomarkers, such as sodium ions (Na^+^) [234]. Several research works focused on correlating stress levels to sweat biomarkers, such as cortisol [129,141], using psychological tests, ice tests, or perceived stress scale tests. However, it was shown recently that sweat stress biomarkers, such as creatinine, could be correlated with human heat stress using a neural network algorithm in which a multimodal sensor could monitor the stress creatinine in sweat and the heart rate and predict the stress intensity occupied by mental states [235]. Figure 7b shows heat-stress creatinine data outputs from the decentralized epidermal sensor, mapping with the neural network algorithm.

In addition to sweat, AI technology and ML techniques were also used to detect glucose levels in artificial saliva (GlucoSense [236]). GlucoSense was a paper-based microfluidic platform that was used to perform a rapid colorimetric evaluation of glucose. It used machine learning and a cloud system to remove the effect of ambient light for real-time image processing using a smart phone to improve the platform against illumination variance and camera optics. Since using AI technology for biosensing is still in its early stages, there is not a great deal of literature on using it for other biofluids, such as tears, saliva, urine, and ISF.

### 4.3. Electrophysiological and Optoelectrical Sensors

#### 4.3.1. Wearable ECG Monitors

Integrating AI and ML algorithms into wearable ECG sensors has substantially enhanced the accuracy of ECG analysis [181]. They also hold the potential to transform cardiac care, promote preventive healthcare, and enhance overall patient well-being [8,20]. For instance, signal processing algorithms for high-resolution ECG sensors significantly improved the fidelity of ECG measurements, enabling early detection of arrhythmias, ischemic events, and other cardiac abnormalities [20]. AI-based algorithms with high sensitivity and specificity can effectively detect cardiac abnormalities, including arrhythmias and heart rate variability patterns. This enabled early detection of potential cardiac conditions, allowing for timely interventions and improved patient outcomes. AI combined with ECG analysis hold promise for personalized healthcare, where treatment plans can be tailored based on individual ECG patterns and risk profiles, leading to more effective and targeted medical interventions [4,8,20].

Motion artifacts that affect signal quality during physical activities are another critical area when using AI with wearable ECG sensors. In this regard, advanced signal processing techniques, adaptive filtering, and motion compensation algorithms have been developed to mitigate these artifacts. Therefore, AI-based ECG monitors can provide more reliable and accurate data, even during dynamic movements [8].

Novel electrode designs and materials have been explored to conform to the skin’s contours while maintaining signal quality, ensuring long-term comfort for users during continuous ECG monitoring. Additionally, multimodal sensing, combining ECG data with other physiological and environmental parameters, such as EOG, EMG, temperature, strain, humidity, and bacterial infection, facilitated a comprehensive health profile [8,181,237] (Figure 7c). Thus, an ANN for these important physiology signatures could make almost individual differences negligible [237].

Knowing that wearable ECG monitors could monitor respiration rate, blood pressure, temperature, and activity levels, AI and ML algorithms could help continuously monitor cardiovascular vital signs to enable early intervention [8,238]. Integration with smartphones and mobile devices enhances the accessibility of wearable ECG monitors for real-time monitoring, data storage, and cloud-based analytics, enabling seamless integration into telemedicine and remote patient monitoring systems [4,16,17].

AI methods are critical for the implementation of biosensors and digital diagnosis. To measure the stress through heart rate and blood pressure, fuzzy-assisted AI Petri net was used to capture time and frequency differences and assess stress levels [196]. Moreover, continuous glucose monitoring is not possible without involving AI in sensors for diabetes management [195]. In addition, AI was employed to analyze data from EEG sensors (brain activity) to assess short-term memory. The results indicate that 90.12% of displayed images were correctly identified, and 90.51% of emotionally responsive individuals accurately answered questions about time and image type [201].

#### 4.3.2. Sleep Monitoring Devices

Sleep monitoring wearable devices have gained significant popularity in consumer health and sleep research in recent years as a non-invasive and convenient tool for tracking sleep patterns and assessing sleep quality [197,239] (Figure 7d). These devices utilize various sensors and algorithms to monitor sleep stages, duration, and disturbances.

Ongoing technological advancements in sleep monitoring wearables have improved accuracy and user experience. These devices now incorporate multi-sensor setups, utilizing accelerometers, heart rate monitors, and peripheral oxygen saturation sensors to capture comprehensive sleep-related data [239]. Advanced signal processing algorithms, including ML and AI, have enabled better differentiation between sleep stages and subtle changes in sleep patterns [240]. The validation studies could align wearable data with gold-standard polysomnography (PSG) in measuring sleep duration and detecting sleep events [241]. Furthermore, sleep data were correlated with various health outcomes, such as cardiovascular health, obesity, and type 2 diabetes [242], highlighting sleep data‘s significance in understanding overall health. In addition, AI can help with automated sleep stage classifiers and EEG pattern detectors with easy access to huge sleep datasets, such as the National Sleep Research Resource [243]. The SLEEPNET algorithm [244], a deep recurrent neural network on EEG signal features derived from routine clinical PSG data, could train on 10,000 PSG recordings from the Massachusetts General Hospital Sleep Laboratory (Figure 7e). Their algorithm reached an overall accuracy comparable to human-level performance of 85.76% (N1: 56%, N2: 88%, N3: 85%, REM: 92%, Wake: 85%). By improving user experience and wearability, with researchers designing lightweight and comfortable devices and providing feedback using AI-enabled sleep monitoring devices, positive and healthy sleep habits can be expected.

#### 4.3.3. Wearable Devices for Mental Health Monitoring

Integrating AI technologies has transformed mental health care, enabling new possibilities for identifying and intervening in mental health issues via immediate and personalized support [186]. Precision therapy and diagnostic systems have contributed to early detection and individualized treatment, leading to more efficient interventions. For instance, Chatbots are highly effective tools for delivering mental health services through digital platforms [186]. However, it is crucial to recognize that AI technologies cannot replace the essential human element of empathy and emotional connection in mental health care.

Implantable sensors represent a frontier in healthcare, designed to interact with biological components for support and treatment of bodily functions and mental health monitoring [245,246]. For instance, lost functions resulting from neural damage can be monitored using neural prostheses. Wang et al. [247] fabricated a spiked ultra-flexible neural (SUN) interface for recording from the peripheral nervous system. This enabled the SUN interface to deform following nerve movements and penetrating spiked electrodes for intra-fascicular recordings. They performed in vivo experiments on the rat sciatic nerve to detect small amplitude electroneurogram (ENG) signals with a high signal-to-noise ratio (SNR). They could differentiate tactile from proprioceptive stimuli and classify the location of the stimulus with a high spatial resolution (Figure 7f). With the current progress in implantable devices for mental health monitoring, AI-enabled implantable sensors will be the future of cognitive and neurodegenerative disease diagnosis.

## 5. Conclusions

### 5.1. Summary of Key Findings and Challenges in AI-Enabled Wearable Health Technology

This review is comprised of two parts. The first part focused on different materials and fabrication methods for developing wearable devices for human health monitoring, including examples of sensing platforms consisting of chemical and physical sensors for monitoring biopotentials (ECG/EMG), biophysical (strain/movement/pressure/temperature), and biochemical (pH/Glucose/lactate). The second part explained the integration of wearable sensors with AI technology, with case studies to show that AI plays a vital role in enhancing the accuracy and reliability of health data captured by wearable sensors.

Wearable sensors encompass diverse forms, such as textile-based, mouthguards, contact lenses, microfluidic patches, and pressure sensors, and are essential in helping healthcare providers perform personalized monitoring of the user physiological and chemical parameters with high accuracy. However, the challenge lies in the electronic readers required to interpret these signals and transmit the data to mobile apps [248,249]. Continuous data reading is imperative for these devices to comprehensively capture an individual’s physiological information over an extended period. Electronic readers for wearable devices must possess long-lasting battery power to sustain continuous data reading. Yet the persistent operation of AI algorithms for data processing can strain battery life, necessitating a delicate balance between sophisticated processing needs and practical battery constraints [248]. The accuracy and reliability of data collected by these wearables are critical, demanding precise sensor calibration, interference minimization, and accounting for variations in user activities and physiology. Calibration and validation processes are crucial to uphold data accuracy [248,249]. In the realm of AI-based data collection for human physiological information, privacy and security considerations are paramount. Wearable sensors are intricately linked to smartphones for seamless data transfer, but the integration of third-party apps on smartphones introduces potential vulnerabilities to data hacking [250]. The prevalent use of Bluetooth, NFC, or Wi-Fi for connecting wearable devices to smartphones poses security challenges, emphasizing the importance of secure wireless connectivity to mitigate the risk of data breaches [250].

AI and Machine learning enable multiple aspects of health status assessment, such as disease prediction and diagnosis, and provide effective treatment. AI-enabled wearable health technology holds tremendous potential to transform the healthcare landscape, revolutionizing how we monitor and manage our well-being. These innovative devices can continuously track various health parameters, giving users personalized insights and feedback [6,12,13,16,188]. As technology evolves, AI-enabled wearable health technology can catalyze a paradigm shift in healthcare towards a more preventive and proactive approach. By offering personalized and continuous monitoring and remote patient monitoring, these devices can help individuals make proactive health choices, preventing potential health complications and reducing the burden on healthcare systems.

Along with the promises of AI in wearables come significant challenges that demand attention and resolution. These devices accumulate a wealth of sensitive data, including heart rate, sleep patterns, and location information. It is imperative to implement robust encryption, data anonymization, and strict access controls to safeguard users’ personal information from potential threats from hackers and malicious actors. Another critical challenge is ensuring the accuracy and reliability of the AI algorithms that drive these wearable health technologies. As these algorithms process vast amounts of data and identify patterns that may elude human observation, subjecting them to rigorous testing and validation is essential. The efficacy of these algorithms in diagnosing medical conditions and predicting health outcomes must be thoroughly assessed to avoid false positives or negatives, which could lead to incorrect medical decisions. Furthermore, considering the diverse nature of human physiology and behavior, it is essential to design AI algorithms that are adaptable and inclusive, catering effectively to various demographics.

Despite these challenges, AI-enabled wearable health technology presents tremendous opportunities for enhancing healthcare by providing real-time feedback. This can empower individuals to adopt healthier lifestyles and be aware of early health issues, such as irregular heart rhythms or sleep disorders, enabling timely interventions and potentially life-saving actions. Moreover, aggregating continuous data from a large population using these wearables offers valuable resources for population health studies and advances in medical research. Such data-driven insights can contribute to a better understanding of health trends, disease patterns, and the effectiveness of interventions.

### 5.2. Opportunities for Future Research and Development

Wearable sensors are the interface to body organs (such as skin, muscles, and eyes) and therefore they must match the mechanical flexibility of human tissues to reduce any negative long-term effects and enable sensing transduction mechanism with high signal-to-noise ratio. In addition, different nanomaterials and fabrication technologies have been used to improve the sensor platforms’ sensitivity, selectivity, and physical and mechanical performance. However, the repeatability, reproducibility, and dynamic stability of sensors remain an issue. The fabrication methods need to be simpler and faster to improve the performance of the wearable sensor, such as reliability, accuracy, high resolution, high detection response, full integration, and mass production. Particularly for biosensors, the stability of sensors in different environments, such as humid environments, long-time use at room temperature, and longevity is important. Although various target biofluids were determined, their body accessibility and ease of collection for continuous and real-time on-body measurement are key issues. For instance, current sweat stimulation methods, including iontophoresis, sauna, and exercise for on-body access or collecting tears, are still uncomfortable for everyone. In addition, despite all the progress made in wearable biosensors, there is a gap in the literature in finding a relationship between biofluid signals and human health parameters [251]. Only a few studies have focused on this, such as one recent work on cortisol sensing and its relation to human stress levels [129] and glucose sensing and its relation to diabetes levels in patients [252], or physiological data related to Parkinson’s disease [253].

AI and Machine learning can define the relationship between health characteristics and bio-signals for future directions. Using AI technology with biosensors is still in its infancy. Thinness, miniaturization, integration and less power-hungry devices are the future of AI-biosensors for healthcare. The current research focuses on using AI technology in biosensing sweat and blood biomarkers. Therefore, it can expand to other biofluid sensing assays such as saliva, ISF, and tears. In addition, it is interesting to correlate chemical signals to the body’s physical/physiological conditions, such as bio-signals to brain activities or body temperature, heart rate, blood oxygen, blood pressure, or brain functions in muscle movements, to obtain complementary health data. In addition to health characteristics, potential disease signals should be considered as part of AI algorithms.

Machine learning and big data processing can help to predict general and complex health conditions. Moreover, to improve the efficiency of disease diagnosis and treatment quality, AI algorithms can be trained with high-quality data, including all the necessary data from wearable sensors and users’ current and previous health and diseases history, to ensure the accuracy of data management and developed patterns. Using the most up-to-date data is also necessary to ensure the latest information goes into the data models. Moreover, to make the technology user-friendly for patients, communication devices or apps need to be affordable and compatible with most smartphones. Miniaturization and lower power consumption can help AI-sensors function more efficiently.

Lower cost, time, and fewer visits are some of the key benefits of using AI-enabled wearable sensor technology in healthcare systems [254]. This improves health management, disease diagnostics, and speed of treatments. Combined wearables, wireless communications, and AI algorithms bring the possibilities of in situ health data analysis, in situ treatment, such as drug delivery systems, and in situ feedback. The feedback can be relative to both treatment effectiveness and the users’ perspectives, which could relate to elderly or young people for regular and personal health monitoring and diagnosis. Therefore, combining AI technologies with wearable health technologies, can provide P4 medicine (predictive, preventative, personalized, participatory) and can provide a new platform for future medical diagnostics in nearly every aspect of healthcare.

## Data Availability

There are no additional raw data for this paper. The paper only uses secondary data from published papers, and all credits for these data have been made via citations and copyright permissions.
